# Reply to Greene: No version of the dual process model can explain rational performance by people who made compromise moral judgments

**DOI:** 10.1073/pnas.2220909120

**Published:** 2023-06-06

**Authors:** Leda Cosmides, María Teresa Barbato, Daniel Sznycer, Miguel Ángel Labarca, Ricardo Andrés Guzmán

**Affiliations:** ^a^Department of Psychological and Brain Sciences, Center for Evolutionary Psychology, University of California, Santa Barbara, CA 93106-9660; ^b^Centro de Investigación en Complejidad Social, Facultad de Gobierno, Universidad del Desarrollo, Santiago 7610658, Chile; ^c^Department of Psychology, Oklahoma Center for Evolutionary Analysis, Oklahoma State University, Stillwater, OK 74078-3064

How does the mind resolve moral dilemmas? We considered two models: Greene’s dual process model (DPM) and our computational model of a moral tradeoff system (MTS) (1). We tested their predictions with a repeated trolley-like dilemma that pits the lives of civilians against the lives of soldiers. Unlike other dilemmas, subjects could choose intermediate options, which sacrifice some (but not all) civilians to save more (but not the most) soldiers. We called these choices compromise judgments.

Greene’s title states his central point: The DPM “does not deny that people can make compromise judgments” (2). But he does not mention (or dispute) the central point of our paper: The DPM cannot produce compromise judgments *that respect the axioms of rational choice*—such as the generalized axiom of revealed preferences (GARP). GARP is an exacting standard of rationality, which applies to a series of judgments made under changing conditions. GARP is satisfied when all these judgments are mutually consistent (p. 7). Rational performance—few if any GARP violations—is a by-product of how the MTS works.

The MTS solves optimization problems. It represents moral preferences via a function, that maps solutions (e.g., save 2M civilians and 3M soldiers) onto levels of moral rightness. Given a set of solutions, the MTS uses this “rightness function” to compute which available solution is *most* right.

When moral incentives change (e.g., the number of soldiers saved per civilian sacrificed), the set of available solutions changes too. But each time that happens, the MTS computes the optimal solution. Always computing the best solution creates a series of judgments that respect GARP—whether these judgments are compromises or not.

That is what we found. Most subjects made judgments that respected GARP, *even when they chose compromise solutions* and *no matter how many compromises they chose*.

We considered many alternative hypotheses: None produced compromise judgments that respect GARP—including the DPM. The DPM can respond to moral incentives, as Greene says (2, 3). But this cannot, by itself, produce rational performance. As we demonstrated (pp. 8–9), responding to incentives produces many GARP violations—not the median of *zero* violations found for subjects who made compromise judgments.

Greene proposed (4, 5) that the prospect of killing innocents activates an “alarm emotion” prohibiting harm—not a “currency emotion” that can be weighed against other values (such as saving the most lives). This implies no compromise judgments (they require a currency emotion). He called for an empirical test (5), which we did: Most people made compromise judgments—many.

Let us assume a different DPM, that permits compromise judgments; Greene cites ref. 6. But this paper on “integrative moral judgment” never discusses compromises. It tests the moral acceptability of *extreme* judgments, especially utilitarian ones. Would compromises result when acceptability is low? Intermediate? How many civilians should die? The model is too underspecified to know; it claims that aversive emotions and utilitarian assessments “are integrated.” This DPM offers no optimization process—no mechanism that produces rational performance when compromises are chosen (pp. 8–10).

No version of the DPM—not ref. 5, not ref. 6—can explain what the MTS model predicts: compromise judgments that respect GARP.
